# Supplementary material for: Are carbon emissions trading and green financial instruments synergistic? -Comprehensive quantitative research based on content analysis
Source: PLoS One. 2024 Mar 7;19(3):e0298601. doi: 10.1371/journal.pone.0298601 (PMC10919601; doi:10.1371/journal.pone.0298601)
Supplement: S1 Data — (ZIP) [file pone.0298601.s001.zip › Data/地级市Z_F工作报告.zip!/║┌┴·╜¡/╦1⁄2╤╝╔╜╩╨/└┤╫╘╣1⁄2╓┌║┼í╛┬φ┐╦╩2╛▌═°í┐_2016.docx]

各位代表：

现在，我代表市人民政府向大会报告工作，请予审议，并请各位列席的同志提出意见。

一、2015年工作和“十二五”回顾

过去的一年，是我市发展极为艰难、任务极为繁重的一年。面对经济下行压力持续加大的严峻形势，在市委的领导下，在市人大、市政协的监督和支持下，我们始终坚定发展信心，主动适应新常态，着力稳增长、促改革、调结构、惠民生、防风险、保稳定，最大限度减小经济下行的冲击和影响。预计全市地区生产总值完成436亿元，比上年增长3%;城镇和农村常住居民人均可支配收入分别达到20963元和12340元，分别增长5%和7%。

(一)全力以赴稳运行。我们把稳运行作为首要任务，保存量挖潜、抓增量扩张，努力维护经济平稳运行。农业生产持续发展。预计一产增加值实现172亿元，同比增长3%。粮食总产稳定在55亿斤以上，实现“十二连增”。建成现代农业示范带7条、高科技示范园区8个。新增绿色、有机食品基地认证面积10万亩。新增规模以上农业龙头企业5家。畜牧业对农业发展的拉动作用明显增强。新增规模养殖场10个，增加值增长4.5%，拉动一产增长1.7个百分点。投资3.4亿元，农田水利工程竣工37项，完成年度计划6项。新增土地流转面积24万亩、农民专业合作社102个。农业机械化率达到93.5%。集贤和宝清两县建立了三级土地交易服务体系。工业经济降幅收窄。加强工业经济运行监测分析和重点骨干企业帮扶，工业经济降幅逐月收窄。预计全市规上工业增加值实现61亿元，下降7%，降幅比上年收窄42个百分点。钢铁和煤化工行业增加值分别增长22.1%和4.7%。扶持企业技改升级，新上技改项目14项。出台支持双矿改革脱困发展8条措施，投入援企稳岗补助资金2.2亿元，帮助企业降本增效，吨煤成本下降65元。为建龙融资5亿元;帮助其纳入省直供电支持范围。推进省内上下游产业对接，帮助企业拓展市场，签订购销合同22项，销售煤炭280万吨、钢材2万吨、水泥5万吨、空心砖1亿块。引导企业资本运作，中创机械、中顺农业成功上市。鼓励企业自主创新，建龙钢铁公司组建了科技产业技术战略服务联盟，龙煤天泰成立了煤化工博士后工作站。实施中小微企业培育计划，新增中小微企业200户。服务业加速提升。预计三产增加值实现170亿元，同比增长8.8%。电商产业取得新突破。中俄国际经贸产业园跨境电商平台加快建设;电商创业园投入使用。腾讯商城、苏宁易购等30多家平台机构和电商落户;培训3000多人，网店发展到2万多家，交易额突破20亿元。旅游、金融和养老产业快速发展。新增国家3A级景区2家，11条线路和5个景区列入省重点推介项目，青山冰雪旅游园开园;全年旅游收入增长26.4%。金融业增加值增长13.2%;金融机构支持地方经济力度加大，存、贷款余额分别增长7%和25.5%。新增“医养结合”养老中心10家，七星老年公寓开工建设。现代物流商贸等新兴业态呈良好发展态势。预计全市社会消费品零售总额实现119亿元，增长8.5%。

(二)坚定不移上项目。扎实开展“产业项目建设提升年”活动。完善项目建设推进机制。通过制定工作方案、量化目标责任、建立考评机制，加大实绩核查、现场考评和通报督查力度，推进产业项目建设。加大外埠招商力度。通过多种方式广泛招商，签订合作项目103项。全市投资500万元以上产业项目开复工88个，完成投资58亿元。新时代水泥余热发电、鑫沙矾渣综合利用、欧蓓莎一期、宝清新农大市场等43个项目竣工;龙煤天泰芳烃、三聚华本LNG项目进入调试阶段;神华宝清电厂、瑞丰腐植酸制肥、中俄经贸产业园、岭东花菇基地等项目加快建设;双矿热电联产项目已获核准。强化园区建设。投资近亿元，完成各级园区基础建设项目19项。市经济技术开发区创业中心主体工程封顶。编制了四区中小企业园规划。全市园区入驻企业217户。全面推进“双深合作”。与深圳市签订了全面战略合作协议，加强了双方交流和项目对接，签订粮食、食品药品、旅游、对俄经贸、承接深圳产业转移以及文化教育等方面合作协议12项，广东天盈塑料管业、深粮中信粮食加工项目已落地。我市58种农产品入驻深圳绿膳谷体验馆，19种农产品在深农“海吉星”商城上架销售，深圳前海“农迈天下”电商在我市建立了绿色食品直供基地。预计全市固定资产投资实现130亿元，同比下降13.3%，降幅同比收窄52.7个百分点。

(三)突出重点优环境。启动“一创两建”活动，推进美丽城乡宜居环境建设。完善交通体系建设。改扩建依饶公路双集段、依宝公路宝清段等国省道5条、农村公路13条。牡鸡七双佳快速铁路前期工作取得实质性进展，双宝高速公路、佳双机场等项目前期工作加快推进。加强城乡基础设施建设。投资9.5亿元，完成基础设施、老旧管网改造等市政工程76项。主城区新城路、一马路、铁西路、建设路、九阳路等12条主次干道改造竣工通车;新建公交港湾16处，改造平交路口10处，完成尖山至岭东公交化改造。改造各类管网232公里。完成寒葱沟净水厂新国标改造并实现局部供水。投资1.5亿元，完成农村基础设施建设项目40项。强化环境专项整治。开展了城区绿化、住宅小区和交通环境整治等专项行动，新增绿地89公顷、清除超高土面积14万平方米;主城区实现垃圾定点定时清运，主次干道机械化清扫率和清雪率达到100%;实行物业管理网格化;改造住宅小区15个，实施住宅楼“平改坡”16栋;编制了主城区城市交通专项规划，增设电子交通监管设施45处。农村新增绿地1千多公顷，重点村屯绿化达标率100%;改造村内道路62.5公里、排水沟1.5万延长米，新建垃圾处理设施41处。改善生态环境。实施污染减排项目41项，取缔燃煤小锅炉45台，淘汰黄标车2469辆。市污水处理二期投入运行，再生水回用工程主体完工。完成水土流失治理36平方公里。造林6.2万亩。单位GDP能耗下降3.7%。

(四)千方百计惠民生。想方设法破解收支矛盾，多渠道筹集资金加大民生投入。全市民生支出77亿元，比上年增长11.6%。改善居民住房条件。煤矿棚改工程竣工住宅7174套，南市区棚改工程全部完工，新回迁居民2100户。采沉区棚改工程新建、回购住宅13254套，回迁居民1400户。城市棚改工程新建住宅2393套。改造农村泥草房、危房4442套。发放廉租房补贴2303万元。提高社会保障水平。创业就业培训1.6万人，成功创业2073人，带动就业9616人。加大援企稳岗力度，全面启动了双矿富余人员组织化分流。全市城镇新增就业2.5万人。推进基本医疗保险市级统筹，定点医疗机构实现住院即时结算;城乡居民全部享受大病保险待遇。城镇职工和居民基本医疗保险最高限额分别提高1万元和5万元。困难群众医疗救助比例提高到70%。新农合报销比例提高5%。城镇职工养老金提高12.7%。城乡低保救助标准分别提高13.6%和29.6%。规范了机关事业单位职工津补贴标准。社区居委会主任待遇每月提高600元。市区供热费每平方米下调2.66元，降幅8.4%;供热期由198天延长到213天。启动了国企厂办大集体改革。强化基本公共服务。县级公立医院改革稳步推进;实行了医疗机构不良积分管理;市新人民医院建设进入了内部装修。投入2178万元，改造校舍28所，完善了10所学校实验室等教学设施;新增省级义务教育标准化学校7所;九年一贯制学校、幼儿园等项目加快推进。市文广中心投入使用;举办了文化大集和文化夜市，群众性文化活动蓬勃开展。省级体育赛事获奖牌23枚。实施“幸福社区”创建和社区建设“双百”示范工程，建设“幸福社区”39个，友谊县富强社区被评为全国和谐示范社区，四方台区振兴中路办事处被评为全国和谐示范街道。整治不规范民办养老机构，利用政府资产建设了市第三老年社会福利院。加强安全稳定。开展“信访百日攻坚”行动，加大信访矛盾排查调处力度，集中化解信访难题，信访总量下降6.5%。严格落实安全生产责任，开展隐患排查治理和“打非治违”专项行动，生产安全事故起数下降14.6%。开展“春雷行动”、“平安冬季百日会战”等专项行动，各类刑事案件发案数大幅下降。

(五)坚持不懈转作风。深入推进作风改进、效能提升，政府自身建设得到加强。强化法治建设。制定了法治政府建设五年规划。自觉接受市人大法律监督、政协民主监督，人大代表建议、政协委员提案办结率100%。建立了政府权力清单、责任清单和政府法律顾问制度。深化各项改革。完成新一轮政府机构改革，市政府撤并行政机构3个，健全了区级食药机构，整合了质检、农产品、食药检测体系。精简审批事项50项，清理比例26%。公安、安监等6家单位实现网上审批。实行了“四证合一”商事登记和企业信用监管制度。建立了联合执法、执法报批的行政执法机制。推行“一口制”收费制度，取消行政事业收费项目13项。改革投资类项目审批流程，推行了“一次告知、并联审批、一图审查”模式。成立了企业投诉举报中心，进一步优化了发展环境。改进工作作风。扎实开展了“三严三实”专题教育。开展了办公用房、公务用车、吃空饷等专项治理。加大审计监督力度，保障了稳增长、促改革、惠民生等政策措施有效落实。加强廉政建设。认真落实党风廉政建设“一岗双责”，完善了权力运行监督和制约制度机制。“三公”经费支出下降9%。加大违规违纪问题查处力度，查处违反中央八项规定人员65人。

各位代表，2015年是“十二五”收官之年。五年来，特别是近两年来，在支柱产业运行艰难、主导产品量价齐降、经济增速大幅回落的困境中，我们综合施策应对经济下行稳增长、千方百计破解收支矛盾保民生，通过全市上下的不懈努力，顶住了压力，经受住了考验。尽管经济受到严重冲击，但推进经济转型的步伐没有停;改善人民生活水平的力度没有降，特别是广大干部群众加快发展的信心和劲头没有减，为我市转型发展夯实了基础、积蓄了动能。经济基础得到夯实。启动了农业综合配套试验区改革，创新经营主体，培育新型合作社995家，农业产业化龙头企业发展到105家，规模畜牧养殖场达到296个，农业产业化进程加快。竣工亿元以上产业项目93项，建龙无缝钢管和煤化工、神华宝清露天煤矿、新时代水泥、龙煤天泰芳烃等一批重大产业转型项目相继建成或投产;全市规上工业企业达到124户，地方煤矿由144个压减到86个，工业产业层次进一步优化。新增大型商贸综合体3家、专业市场7家、物流企业179家。社会消费品零售总额年均增长12.8%。商贸综合体、现代物流、电子商务等新兴业态快速发展，互联网化推动了传统服务业向现代服务业转型升级。三产增加值年均增长4%，占GDP比重提高了14个百分点。政策支撑更加有力。我市被纳入全国资源型城市可持续发展规划、东北老工业基地搬迁改造规划、全国第三批资源枯竭型城市和“龙江丝路带”哈佳双同产业集聚带。多年谋划牵动全市发展大局的重大问题取得实质进展，市经开区晋升为国家级经济技术开发区，钢铁冶金园区被确定为省新型工业化示范园区，中俄国际城列为省“对俄重点开发开放试验区”，牡鸡七双佳快速铁路、双宝高速公路、佳双机场等一批重大基础设施项目将在“十三五”时期开工建设。城乡面貌显著改观。新建了滨水北城和南市新城，主城区面积由15平方公里扩大到31平方公里。投资36亿元，建设国省道74公里、市政道路160公里、农村公路193公里。投资130亿元，实施了安邦河治理以及路网、场馆、“三供两治”等基础设施项目200余项。主城区物业管理覆盖率、集中供热普及率、污水处理率分别达到95.7%、80%和78%。投资8.4亿元，实施农村基础设施建设项目7000余项，建设农村道路1667公里、饮水安全工程205项。农村自来水通村率达到100%。城乡管护体系逐步完善。人民生活进一步改善。城镇和农村常住居民人均可支配收入分别年均增长7.4%和12.9%。投资80亿元，建设各类棚改工程住宅7.6万套，改造农村泥草房4万户。发放廉租房补贴1.3亿元。城镇人均住房面积提高了26%。新建和改扩建黑煤职院、新一中以及标准化义务教育学校118所、公办幼儿园48所;义务教育普及率达到99%。新建市人民医院、4个县级医院、147个乡镇和村卫生室。社会保障水平大幅提升。全市新增城镇就业13.8万人。企业养老、居民养老、失业、医疗、工伤和生育保险参保人数分别达到11.5万人、24.2万人、12.2万人、54.4万人、8.5万人和6.5万人。6.8万破产、困难企业和厂办集体退休人员纳入城镇职工医疗保险。城镇职工和城乡居民养老金分别提高81.3%和27.3%。实现了医疗保险市级统筹，城镇职工和城乡居民基本医疗保险最高限额分别提高90%和75%。新农合待遇提高2倍。城乡低保救助标准分别提高1.2倍和1.9倍;五保集中和分散供养标准分别提高1.7倍和1.5倍;医疗救助比例提高20%，重特大疾病救助限额提高2万元。

各位代表，成绩来之不易，这是省委、省政府和市委正确领导的结果，是市人大和市政协监督支持的结果，是全市上下拼搏实干的结果，凝聚了全市人民的辛勤汗水。在此，我代表市人民政府，向全市人民，向各级人大代表、政协委员，向各民主党派、工商联、无党派人士和人民团体，向驻双解放军、武警官兵和公安干警，向在双中省直单位和所有关心支持双鸭山发展的各界人士，表示衷心的感谢!

在肯定成绩的同时，也必须清醒地看到，尽管我们付出了很大努力，化解了诸多不利因素，部分指标恢复了增长态势，但总体仍不够理想，不少指标没有达到年初预期，特别是与“十二五”目标相比差距较大，个别指标甚至回落到“十一五”末期水平，暴露出我市经济社会发展中的一些深层次矛盾和问题：一是经济增长动力不足。一产比重过大，市场化程度低;生产性服务业发展较慢，三次产业融合度偏低。双矿、建龙等重点企业效益下滑严重，地方煤矿、粮食加工企业开工不足，企业竞争力不强，尤其是重点产业项目多处于建设期，经济增长动力转换不够。二是产业转型步伐不快。存量改造升级缓慢，扩大增量调整结构成效不明显，产业依然偏重资源和传统，原字号、粗加工产品比重过大，对经济增长的拉动力有限，转型升级任重道远。三是财力保障难度加大。财税大幅减收，全市公共财政预算收入仅完成18亿元，回落到2009年水平，而保工资、保运转、保民生等刚性支出是2009年的2倍多，特别是多渠道筹资长效机制尚未建立，政府偿债压力加大，收支矛盾突出。四是民生改善任务艰巨。新人民医院、学校等重点民生工程还没有如期完工，棚改、供水、供气、空气质量、城市交通、公共服务等方面与群众要求仍有很大差距。五是社会不稳定因素增多。由于企业开工不足，效益下降严重，职工稳岗压力加大，就业再就业形势严峻;企业职工收入水平受到不同程度影响，部分群众生活出现困难，加之棚改、国企改革触及诸多历史遗留问题，社会不稳定压力增大。以煤矿和化工为重点的安全生产形势严峻。六是政府自身建设仍需加强。我市经济社会发展中产生的矛盾和问题，既有客观因素，也有我们工作不到位的原因，主要表现在政府职能转变滞后，审批效能不高、发展环境不优等问题还比较突出;一些干部思想观念落后、改革创新意识不强、破解难题能力不足，特别是面对经济下行压力，缺乏勇于担当、攻坚克难的责任感，回避矛盾和困难，为官不为、懒政怠政等问题还不同程度存在。对此，我们一定高度重视、采取有力措施，认真加以解决，决不辜负全市人民的期望和重托。二、“十三五”时期和2016年工作总体安排

各位代表，“十三五”时期是我市全面建成小康社会的决胜时期，也是加快转型升级的关键时期。我们面临的形势错综复杂，经济新常态下矛盾愈发凸显，不可预见因素增多，下行压力依然较大，稳增长调结构惠民生难度加大，转型升级、爬坡过坎任务异常艰巨，未来依然充满挑战。必须充分认清，我们遇到的困难，表象在于宏观经济形势冲击所致，经济发展新常态下，“三期叠加”矛盾集中凸显，形成了经济持续下行的巨大压力。根源在于我市长期积累的结构性、资源型、体制性“三大矛盾”相互交织，面对经济下行的冲击集中迸发。核心在于创新驱动的发展方式和新的发展动能尚未形成，资源、绿色和生态优势没有转化成经济优势，经济总量不大、质量不高。更要充分看到，有利因素依然很多，总体向好的基本面没有改变，支撑转型发展的要素正在聚集，我市处在“龙江丝路带”战略节点上，国家“一带一路”和“龙江丝路带”建设规划的深入实施，国家和省为支持煤城脱困转型发展量身定制的特殊政策正在持续发力，特别是国家全面实施供给侧结构性改革将释放巨大的政策“红利”，我市资源、绿色和生态比较优势突出，传统产业改造提升和新兴产业培育壮大空间巨大，转型发展的机遇千载难逢。我们必须增强忧患意识、机遇意识和创新意识，主动适应和引领经济发展新常态，在困难中争取主动、增强定力，用决心、信心和行动，让双鸭山在新常态下迈上新台阶、实现新突破。

市委十届七次全会提出的“十三五”时期经济社会发展总体思路是：按照“五位一体”总体布局和“四个全面”战略布局，牢固树立和贯彻落实“五大发展理念”，坚持发展是第一要务，坚持稳中求进，坚持转变发展方式，主动适应和引领经济发展新常态，围绕全省深入实施“五大规划”和城市转型发展主线，全力打造现代煤电化钢产业示范区、绿色有机食品产业聚集区、黑土湿地旅游核心区和对外贸易服务区，着力完善体制机制，着力推进结构调整，着力鼓励创新创业，着力保障和改善民生，统筹推进经济建设、政治建设、文化建设、社会建设、生态文明建设和党的建设，奋力实现全面建成小康社会的宏伟目标。

主要预期目标是：到2020年，全面建成小康社会。综合实力迈上新台阶，地区生产总值年均增长6%以上，规模以上工业增加值年均增长15%左右，固定资产投资年均增长10%以上，公共财政预算收入年均增长6%，社会消费品零售总额年均增长8%。结构调整实现新突破，三次产业排序调成“三、二、一”。城乡面貌呈现新变化，市区集中供热普及率90%以上，全市生活垃圾无害化处理率92%，主城区污水收集率95%以上。人民生活达到新水平，城镇和农村常住居民人均可支配收入分别年均增长7%和5%，基本完成棚户区和农村危房改造。生态环境得到新提升，安邦河达到Ⅳ类水体水质标准，乌苏里江双鸭山段干流达到Ⅲ类水体水质标准，主要污染物排放量显著下降，城市环境空气质量达标率88%。社会和谐开创新局面，整体达到省级文明城市水平，争创“全国文明城市”。

2016年是我市全面建成小康社会决胜阶段的开局之年，也是推进结构性改革的攻坚之年。市政府工作的基本要求是：深入贯彻落实中央和省委经济工作会议精神，牢固树立和贯彻落实“五大发展理念”，适应经济发展新常态，落实市委十届七次全会的部署，以资源型城市转型为主线，着力加强结构性改革，去产能、去库存、去杠杆、降成本、补短板，提高供给体系质量和效率，提高投资有效性，加快培育新的发展动能，着力稳增长、调结构、惠民生、防风险，综合施策，扎实起步，努力实现“十三五”时期全市经济社会发展的良好开局。主要预期目标是：地区生产总值增长4%左右;规模以上工业增加值增长1.5%;公共财政预算收入增长3%;固定资产投资增长8%以上;社会消费品零售总额增长8%;外贸进出口总值增长3%;城镇和农村常住居民人均可支配收入分别增长7%和5%;节能减排完成省下达指标。三、着力构建新型产业体系

按照市委提出的建设“四大产业区”战略目标，坚持绿色高效发展，着力实施创新驱动，以发展现代煤电化和冶金循环产业为重点，加快建设现代煤电化钢产业示范区;以发展优质高效现代农业和绿色食品加工产业为重点，加快建设绿色有机食品产业聚集区;以发展北大荒黑土湿地旅游产业为重点，加快建设黑土湿地旅游核心区;以发展外向型产业为重点，加快建设对外贸易服务区;大力发展现代服务产业，助推“四大产业区”建设，全面推进结构性改革，努力构建新型产业体系，促进全市经济转型升级。

(一)加快优质高效现代农业发展。“十三五”时期，以绿色高效为目标，以“调结构、抓有机、走高端、创品牌”为路径，推进农业结构向以“两牛一猪多禽”为重点的畜牧业调整，种植结构向以马铃薯、红小豆、白瓜籽以及林中产品等为重点的经济作物调整，粮食结构向以优质水稻、特色玉米和非转基因大豆等为重点的优质高效作物调整，以“互联网+”为手段，推进“种养加销”一体化发展，实现“种得好”向“卖得好”、“种得更好”转变。2016年，以提质增效为目标，着力“增水稻、调玉米、稳大豆、扩杂粮、强果蔬”，经济作物比例提高3个百分点。扶持谷实种猪，壮大安信牛业，通达和七星牧业扩大到万头规模，建设一批新的规模养殖场，奶牛存栏和肉牛、生猪、家禽出栏分别增长7%、4%、5%和2%。推进农业“三减”行动，建设绿色有机食品基地39万亩;有机食品基地1个、绿色食品基地56个。每个县新建规范化合作社50个、典型合作社2个、产加销一体化经营合作社2个。四个县农产品质量检验检测中心投入使用。推进“互联网+农业”，建立农产品质量追溯、质量监管、市场监测预警和病虫害监测预报系统平台;借助淘宝、腾讯、百度等电商平台，打通农村电商全渠道，打造农业电商产业链。

(二)加快现代煤电化产业发展。“十三五”时期，坚持“绿色高效安全”发展，加快国有大型煤矿技术改造、降本增效和地方煤矿整治整合，推进现代化矿井建设;以“低碳、环保”为目标，加快煤电产业转化升级，发展风电、太阳能和生物质发电等清洁能源产业;通过以资源换项目、煤矿联项目的方式，吸引和聚集新的煤化工资源，延伸煤炭产业链条，开发煤制芳烃、煤制烯烃、煤制天然气、煤炭地下气化、煤炭间接液化等现代煤化工项目，推动采掘产业现代化、电力产业清洁化、化工产业高端化发展，全力建设国家级煤化工产业示范基地。2016年，以创新扩量为目标，抓住我省淘汰劣质煤的机遇，扶持煤炭企业扩大域外市场。推进结构性改革政策落实，完成国有七星煤矿破产，加快地方煤矿整治整合，积极稳妥化解低水平产能;深化国有煤矿改革，降低吨煤成本。确保三聚华本LNG、龙煤天泰10万吨芳烃、神华宝清120万千瓦电厂等项目投产;力争龙泰60万吨煤制烯烃、双矿2×35万千瓦热电联产、华本费托合成油、宝山煤炭地下气化等项目开工建设。加快北京神雾聚乙烯、神华宝清褐煤提质、大唐热电 二期、东荣三矿扩建等项目前期。

(三)加快冶金循环产业发展。“十三五”时期，以“强基固本、高端开发、循环发展、规模扩张”为主攻方向，推进建龙钢铁降本增效、升级改造，大力开发高端钢材、新型管材、板材和金属制品等高端产品;继续延伸冶金废弃物循环利用产业链，推进余热余压、固体废物和废水资源化利用，强化节能减排治理，积极开发新型建材、焦炉煤气和粗苯等项目;助推装备制造业发展，实现冶金产业循环发展、转型升级;利用境外资源，推进规模适度扩张。2016年，以激活存量为目标，继续加大扶持力度，引导和促进建龙钢铁活化经营机制、强化管理、降本增效，实施资本运作，推进股份制改造和多渠道融资，在行业重组中站稳脚根。抢抓国家支持重大基础设施建设的机遇，帮助企业拓展市场，扩大产品地方销售比例。加快建龙干熄焦项目建设，新时代水泥废渣综合利用、油气钻井套管机械加工项目竣工。支持新时代水泥发展特种水泥等新型建材。推进石墨、玄武岩等非金属矿产资源开发利用。

(四)加快绿色食品加工产业发展。“十三五”时期，突出绿色兴市、绿色富民，以绿色农业、林中经济和北药为基础，以“真绿、安全、美享、时尚”为主攻方向，坚持“高端化生产、品牌化推进、特色化支撑”，以粮产品、畜产品、菌产品、山产品、果菜产品、水产品、蜂产品、药产品八大“真绿”产品为重点，大力发展绿色有机食品加工和生物制药产业，培植八大类“真绿”产品基地，提高绿色有机食药产品供给能力，推动生态优势向产业优势、经济优势的转变。2016年，以培育新动能为目标，通过引大联强、资产重组，推进工艺、产品改造升级，激活传统农产品加工企业，力争一批农产品加工企业实现重组，加大力度引进项目，扶持绿色有机食品示范企业发展，打造高端食品加工企业。加大绿色有机食品和地理标识认证力度，建立绿色有机食品质量追溯机制，确保“真绿”品质。加大品牌创建和宣传推介力度，发挥互联网融合优势，叫响我市“真绿”品牌。加快吴越农业科技园等项目建设;宝清滑膜南瓜籽油、东北大自然米糠提取等项目竣工;力争远东150万头生猪屠宰加工、黔牧万头肉牛养殖及加工等项目开工建设。推进食品药品产业园区建设，大力招引国内外知名制药企业，力争在制药产业发展上有突破。

(五)加快北大荒黑土湿地旅游产业发展。“十三五”时期，充分发挥我市独有的北大荒品牌资源优势，以发展绿色农业游、湿地生态游、乌苏里江风情游和山水城市近郊游为主攻方向，坚持开发与保护并重，优化产业布局，打造精品景区，创新运营模式，强化市场开拓，完善服务功能，促进互联网与旅游业高度融合，增强生态旅游产品供给能力，打造“北大荒黑土湿地之都”品牌。2016年，以激活要素为目标，深入挖掘北大荒自然旅游资源文化内涵，推动旅游与文化创意等产业融合发展，加快服务配套，提升品质，打造精品景区。引进战略投资者开发旅游资源，创新经营模式，提升经营水平，打造市场化经营主体，建立面对域内外的大经营体制。整合现有5条精品线路，利用途牛网、深圳商报、世界之窗等多种方式，做好旅游推介。推进旅游企业与美团网、糯米网等网络媒体对接合作。加大与深圳市旅游合作力度，做好赫哲民俗文化对外展示。围绕组团、自助、自驾等旅游方式，运用线上线下服务方式，建设市域综合旅游服务体系。发挥联席会议、行业协会作用，强化住宿、餐饮、购物、客运等优质旅游服务单位配套服务。推进七星峰等旅游景区道路建设，增强可进入性。

(六)加快外向型产业发展。“十三五”时期，以“出口抓加工、进口抓落地”为主攻方向，以主城区为核心，以饶河县为先导，辐射市域周边六个口岸，以7个出口基地为支撑，发挥通道作用，实施“龙江丝路带”哈佳双同产业集聚带建设，实现双向开放、产业聚集，形成以对俄开放为重点，面向日韩、欧美等国家的全方位、多层次开放新格局，为城市转型助力。2016年，以开放融合为目标，充分利用招商线索，加大对接洽谈力度，加快项目转化落地进度。推进中俄国际经贸产业园、饶河互市贸易区建设，提升四达国际贸易功能，打造以商贸为龙头，集贸易服务、产品加工、国际物流、国际结算和跨境电商于一体的贸易综合体。推进中俄国际经贸产业园保税区申报工作。力争中俄国际经贸产业园结算中心列为全省试点。推进饶河口岸联检大厅和四达交易中心监管库建设。力争中俄经贸产业园货运枢纽中心、配送中心和冷链物流等项目投入使用。大力扶持桦宝、和兴等出口创汇型企业，积极引进大集团从事跨境投资合作。推进中俄国际经贸产业园进出口加工区、建龙钢材制品、宝清白瓜籽杂粮、饶河绿色蔬菜、岭东花菇等进出口加工基地建设。通过跨境电子商务、组织经贸活动，加强线上线下对接，扩大贸易规模，提高铁精粉、食品进口量，增加果蔬、肉类和建材等地工产品出口比重。

(七)加快现代服务业发展。“十三五”时期，以便利、诚信为宗旨，以“互联网+”为主线，以新业态、新模式为途径，由服务域内向服务域外拓展，大力发展现代商贸物流、现代金融信息、电子商务、健康养老、家庭服务等新兴服务业，推动现代服务业提速升级，增强服务供给引导消费。2016年，以发展新业态、新模式为目标，改造提升传统商贸物流产业，加快大型批发市场、物流等流通体系建设，大润发、皇都等项目投入运营，联丰国际城市综合体项目加快建设，南市区、滨水北城等配套商服开工建设。完善4G覆盖和城域网等信息设施，建设4G网络基站529个。引导加工和商贸线上线下深度融合，扩大市场份额。拓展与深圳农产品交易中心合作，推进线上线下产销对接联动，拓展域外市场。推进与阿里巴巴深度合作，建设各级电商运营中心、物流中心和村级淘宝服务站，实现“网货下乡”和“农产品进城”双向流通。发挥电商创业园培训、示范和孵化作用，创建产业园和示范园，增强电商发展和互联网应用承载能力。壮大金融产业，力争引进金融机构1家，农信社改制为农商行1家;提高证券业、保险业财政贡献率;搭建产业发展引导基金投融资平台。促进金融机构扩大信贷规模，支持重点企业、项目和中小企业、三农经济发展。鼓励直接融资，加大在多层次资本市场融资力度，力争再上市1-2家企业。加强金融诚信建设，打击恶意逃债，防控金融风险。金融机构贷款余额增长7%以上。打通供需通道，促进化解商品房存量与保障房安居工程建设有效衔接，充分释放住房刚性需求和改善性需求，化解房地产库存。鼓励和引导社会力量投资开发候鸟式养老、健康养老等养老产业项目。加快家庭服务业发展。四、全力加快宜居城市建设

坚持“统筹、共享、文明”城乡建设理念，树立以人民为中心思想，加快提升宜居环境质量和民生改善水平，最大限度让双鸭山百姓共享改革发展成果。

(一)加强基础设施建设。“十三五”时期，以补齐城乡基础设施短板为重点，加快铁路、公路、机场等重大交通设施建设，加强市政路网、“三供两治”基础设施建设，推进流域治理、灌区改造等水利设施建设，构建适度超前、功能配套、科学高效的现代化城乡基础设施体系。2016年，推进重大交通设施建设。加快西外环路、依饶公路集太段建设。集当公路尖山至宝山段、饶盖公路饶河口岸至大岱林场段、饶河客运站开工建设。完成依饶公路、富密公路局部改造。力争牡鸡七双佳快速铁路开工建设。饶换铁路、双宝高速公路、佳双机场等项目加快前期工作。推进城乡基础设施建设。加快主城区道路建设，打通电大路，升级南环路，改造九阳路，实施新兴大街、东西平行路交通渠化改造，改造升级背街巷道23条，打造微循环交通体系。加快县域城关镇和老矿区主次干路改造，完善市政设施配套。加快市经济技术开发区等重点园区基础设施建设。系统性改造主城区供水干线和小区老旧管网，主城区和岭东区实现24小时供水。新建东保卫、安泰、扩建新安、双阳矿净水厂。改造主城区老旧供热管网。启动新气源建设，扩大入户率，提高供气质量。推进农村道路硬化、沟渠改造、村屯亮化，以及供水、垃圾处理设施建设。推进水利基础设施建设。实施安邦河市区段治理、寒葱沟水库清洁小流域水土保持、饶河大岱河和小佳河、四排灌区改造和矿井水综合利用工程。推进“三江连通”项目前期工作。

(二)推进“一创两建”。“十三五”时期，以“一创两建”为牵动，坚持“科学统筹、建管结合、注重特色、生态宜居”，持续推进城乡环境治理，创新城乡环境管护和社会治理，提升城乡环境质量和社会文明程度，打造优美整洁、安全有序的和谐宜居城乡环境。2016年，推进城乡环境整治。继续实施城镇绿化和市容环境、住宅小区、交通环境整治，启动集贸市场整治行动。主城区改造老旧小区15个;规范建设中心区停车场16处;辟建农贸市场4处;增设电子交通监管设施91处。加大环卫保洁和冬季清雪力度，提高道路机械化湿式清扫率。狠抓乱贴乱画、乱搭乱建，清理违法经营摊点。搞好村屯绿化美化、村容环境整治和改厕改灶。强化城乡管理。推进“多规合一”，编制海绵城市规划。实施主城区交通专项规划控制和管理，出台城市建设新规，强化城市规划、建设和管理工作。推行日常保洁、绿化养护、市容监管、市政设施维护“四位一体”环境长效管护机制。完善物业网格化管理，力争主城区物业管理全覆盖，提高物业服务水平。进一步推进精细化城乡管理体系建设。力争全市60%以上社区建成幸福社区。加强社会治理。树立隐患就是事故的理念，以煤矿和化工安全为重点，严格落实企业主体责任，加大隐患排查整改和安全监管力度，强化过程责任追究，对存在安全隐患的单位及人员严肃问责，坚决杜绝重特大事故发生。进一步加强和改进信访工作，有效化解各类社会矛盾。加大“平安双鸭山”建设力度，推进实战化警务改革，完善立体化治安防控体系，保持严打各类犯罪高压态势。做好第五届社区居委会换届选举工作。

(三)强化生态文明建设。“十三五”时期，始终守住生态底线，坚持绿色发展理念，实施“治水、净气、护绿”专项行动，加快构建生态屏障，加大污染防治和节能减排力度，绿色高效节约开发利用资源，执行最严格的生态环境保护制度，建设生态文明示范区，为百姓打造生态宜居美好家园。2016年，推进生态建设与保护。造林7.5万亩，绿化村屯80个。做好双鸭山主峰等6个土地整治和地质环境治理项目申报。力争四个县晋升为国家级生态县，饶河县晋升为国家级生态文明建设示范区。继续加强安邦河流域水环境治理和饮用水水源地保护，实施集中式饮用水水源地保护区环境整治，加快寒葱沟水库水源地治理二期和中水回用工程建设。加强污染防治。推进资源节约利用、低碳排放。确保市污水处理一期和友谊、宝清、饶河3个县污水处理厂稳定运行，市污水处理厂二期和四方台污水处理厂形成减排能力。加快淘汰燃煤小锅炉和黄标车;严控秸秆焚烧，搞好综合利用;加强扬尘和烟粉尘治理。稳妥推进资源类民生价格改革。加强生态环境监管。落实各级政府生态保护主体责任，启动生态责任审计。全面强化执法监督和问责，确保最严格环保、水资源和节约用地等制度落到实处。建立违法企业“黑名单”制度，严厉打击恶意违法企业。

(四)提升社会保障水平。“十三五”时期，从解决群众最根本利益问题入手，以创业就业、住房改善、社会保障和救助为重点，坚持“补短板、保基本、全覆盖、提水平”，逐年提高民生投入，扩大创业就业群体，改善群众居住条件，提高社会保障和救助水平，增强人民群众获得感。2016年，促进创业就业。实施大众创业三年行动计划，推进创业孵化器、创业市场、创业园区等创业基地建设，设立创业基金，实施小额贷款担保、资金补贴等扶持政策，确保成功创业1500人，带动就业8000人;扶持300名高校毕业生成功创业;加大就业扶持力度，新增城镇就业2万人。加大援企稳岗力度，完成双矿转岗分流人员组织化安置。改善住房条件。煤矿棚改工程竣工住宅5000套，上半年完成南市区居民回迁;城市棚改工程新建住宅2192套;采沉区棚改工程建设住宅14514套。积极谋划申报新的棚改项目。改造农村泥草房危房7120套。发放廉租房补贴10500户。提高保险待遇。扩大城镇职工和居民“五项”保险覆盖面，推进机关事业单位养老保险改革，力争事业单位工伤险全员参保。按政策提高各项保险待遇和低保救助水平。城镇职工大病医疗保险最高限额提高1万元，城镇职工养老金提高10%，新农合人均筹资标准提高到560元。力争第三社会福利院二期、老年精神病人福利院开工建设。

(五)协调发展社会事业。“十三五”时期，以加快科技转化、推进教育优质公平、深化卫生体制改革和文体事业繁荣为重点，持续推进基本公共服务设施建设，完善社会服务功能，提升公共服务水平，促进各项社会事业协调发展。2016年，推进科技创新。加快科技公共服务平台建设，新建技术创新联盟2个，完成黑龙江煤化工技术研发中心、饶河农业园区省级园区申报工作，加快国家煤及煤深加工产品质量监督检测中心建设。深入实施科技型企业三年行动计划，转化高新技术成果90项，新增规模科技型企业5户。市经济技术开发区科技孵化器投入使用。利用好结构性改革政策，扶持企业技术改造和产品更新，鼓励工人“小改小革、小发明、小创造”。创新人才培养、引进和留住使用机制，围绕重点产业发展需求，加大科技人才、高端人才和创新团队柔性引进力度，大力培养本地实用技术人才，培育新的发展动能。推进优质公平教育。调整教育网点布局，全力加快义务教育标准化学校建设，四区和宝清县通过义务教育基本均衡县区验收，集贤、友谊和饶河县全面完成义务教育标准化学校建设任务。滨河学校新教学楼、铁西小学开工建设;五中、三十七中、实验学校，尖山光明、岭东树人和四方台育红小学投入使用。新建、改扩建公办幼儿园8所。加大教育“三乱”整治力度，建立师德师风考评长效机制，实行“师德一票否决制”。整合职业教育资源，构建以就业为导向的现代职业教育体系。推进健康双鸭山行动。深化县级公立医院改革，巩固基本药物制度，抓好分级诊疗、双向转诊和医疗联合体建设。完善公共卫生服务体系，落实疾病应急救助制度，提升疾病防控服务能力和水平。全面落实二孩政策。启动市传染病医院异地新建，市新人民医院、市精神病防治院建成。完善食品药品监管体系，保障人民群众生命健康。发展竞技体育，开展全民健身运动，增强人民体质。推进文化繁荣。完善文化基础设施，加快现代公共文化服务体系建设，推动公共文化服务均等化。蓬勃开展群众文化活动，创建地方特色文化品牌。继续做好双拥工作，关心支持妇女儿童、老龄、残疾人等事业，促进民族宗教、外事侨务等事业发展。

(六)做好扶贫开发工作。“十三五”时期，突出“精准扶贫、精准脱贫”，以农村、矿区贫困群众脱贫为重点，坚持开发扶贫与保障兜底双轮驱动，政府、市场、社会“三位一体”推进，实施产业扶贫、创业扶贫和保障贫困群众基本生活条件“二加一”扶贫攻坚行动。2016年，推进创业扶贫。扶持矿区和农村、城郊贫困群众发展特色种养殖、家庭服务和电子商务，促进增收脱贫。突出产业扶贫。以整村(社区)推进为重点，制定连片开发产业扶贫规划，确定重点扶贫产业，实现区域性和群体性增收脱贫。强化政策扶贫。加大农村和矿区基本生活设施建设和改造力度，改善群众生活条件。引导生存条件恶劣地区困难群体有计划外迁。加强医疗、卫生、教育等救助力度，促进贫困地区基本公共服务均等化。探索保障扶贫。推进扶贫开发与低保救助衔接，逐步将无业可扶和无力脱贫的“两无群体”纳入社会救济范围。五、着力强化措施保障

完成“十三五”和今年的目标任务，必须完善制度机制，提升推进落实的能力和水平，为全面建成小康社会提供有力保障。

(一)完善推进机制。建立包保推进机制，对“十三五”规划各项任务实行领导牵头抓总、分级包保推进。建立抓落实责任链条和闭环督办制度体系，制定“任务书、路线图、时间表”，对重点工作全程跟踪、节点督办、限时问效。突出用项目化方式抓发展，把确定的目标任务量化为一个个项目，建立项目化方式抓发展的考评细则和包保推进办法，加大实绩核定、现场考评、定期通报力度，推进工作落实。

(二)深化重点领域改革。推进行政审批制度改革，精减审批项目和流程，完善责任和权力清单，建立负面清单;启动强区扩权、强镇扩权改革;推行网上审批，建立网上审批效能评价和投诉机制;进一步完善市行政审批中心功能和服务网络建设;推行重点领域联合执法。深化行政收费制度改革，精减收费项目和频次。推进税费制度改革，落实结构性减税和普遍性降费政策，依法依规征缴税费，降低企业生产成本。改革预算管理制度，推进政府性基金、国有资本经营预算与一般公共预算统筹，启动中期规划管理。设立产业发展基金、中小微企业担保基金，发挥政府资金引导和放大作用，扶持产业和企业发展。构建多元化投融资体系，推进政府与社会资本合作PPP项目建设;加快政府债务置换，建立偿债准备金，促进投融资一体化。推进市场化配置资源机制改革。抢抓国家供给侧结构性改革机遇，全力做好向上争取政策工作。完成国企厂办大集体改革。启动公车改革。

(三)提升统筹发展水平。深化“双深”合作，加大对接力度，与深圳市在粮食产销、绿色食品加工、旅游、文化等重点领域合作上有突破性进展。坚持城区建设一体规划、基础设施同步建设、产业错位差异发展，加快岭东、四方台、宝山区转型替代产业发展，统筹城区协调发展。深化市县区与双矿、农垦、森工合作，推进在现代煤电化、绿色有机食品、旅游和现代服务业等产业方面紧密合作，推动区域统筹发展。

(四)加强法治政府建设。深入落实法治政府建设五年规划，提升各级干部运用法治思维和依法办事能力，依照法定权限和程序履行政府职能。自觉接受人大法律监督和政协民主监督，认真办理议案、建议和提案，积极听取社会各界意见和建议。严格执行重大行政决策程序规定，提高依法决策水平。健全政府信息发布和办事公开制度，扩大公开领域和事项，重点推进公共财政预算、公共资源配置、公共设施建设等领域政府信息公开。加强公共法律服务体系建设，营造良好法制环境。

(五)转变工作作风。巩固“三严三实”专题教育成果，健全改进作风长效机制。牢记为民宗旨，健全密切联系群众制度机制，市政府班子成员带头深入基层和群众，倾听民意、了解民愿，集中财力办好民生实事。畅通民意诉求渠道，办好市长热线、市长信箱和行风热线，及时解决群众诉求和困难。认真落实首问负责、服务承诺、限时办结等制度，全面推行一线工作法，提高服务效能和水平，促进发展环境优化。新常态下，政府系统干部要保持换挡降速的定力，消除结构调整的顾虑，摆脱传统路径的依赖，转变思想观念，增强转型发展的信心和勇气。政府各职能部门要切实履职尽责，做好全区域指导和服务工作。加大治庸治懒治怠问责力度，坚决纠正庸政懒政怠政行为。

(六)加强廉政建设。严格履行“一岗双责”，深入推进政府系统反腐倡廉体系建设。强化干部廉政教育，严守《准则》和《条例》。深化廉政风险防控，推进公共资金、国有资产、重大项目和领导干部履行经济责任情况审计全覆盖;强化行政监督，在国有资产监管、政府投资和采购、公共资源转让和工程建设等重点领域和关键环节，排查廉政风险点，完善监管制度，严控严管权力运行。坚持勤俭办事，狠抓降本节支，严控“三公”经费支出。强化行政问责，严肃查处各类违纪违法案件，坚决查纠损害群众利益突出问题，保持严查严惩腐败高压态势。

各位代表，市委十届七次全会已经确定了我市未来发展战略。新常态赋予新使命，蕴含新机遇。实现经济转型发展、民生持续改善、社会安定和谐，需要我们携手共建、合力向前。今天的双鸭山，已经站在了新的历史起点，让我们在市委的坚强领导下，务实苦干、勇于担当，锁定目标、奋力前行，为加快推进双鸭山转型发展、全面建成小康社会而努力奋斗!
